# Homing endonucleases from mobile group I introns: discovery to genome engineering

**DOI:** 10.1186/1759-8753-5-7

**Published:** 2014-03-03

**Authors:** Barry L Stoddard

**Affiliations:** 1 Division of Basic Sciences, Fred Hutchinson Cancer Research Center, 1100 Fairview Ave. N. A3-025, Seattle, WA 98109, USA

**Keywords:** Homing endonuclease, Meganuclease, Group I intron, Gene targeting

## Abstract

Homing endonucleases are highly specific DNA cleaving enzymes that are encoded within genomes of all forms of microbial life including phage and eukaryotic organelles. These proteins drive the mobility and persistence of their own reading frames. The genes that encode homing endonucleases are often embedded within self-splicing elements such as group I introns, group II introns and inteins. This combination of molecular functions is mutually advantageous: the endonuclease activity allows surrounding introns and inteins to act as invasive DNA elements, while the splicing activity allows the endonuclease gene to invade a coding sequence without disrupting its product. Crystallographic analyses of representatives from all known homing endonuclease families have illustrated both their mechanisms of action and their evolutionary relationships to a wide range of host proteins. Several homing endonucleases have been completely redesigned and used for a variety of genome engineering applications. Recent efforts to augment homing endonucleases with auxiliary DNA recognition elements and/or nucleic acid processing factors has further accelerated their use for applications that demand exceptionally high specificity and activity.

## Introduction

Homing endonucleases, also termed ‘meganucleases’, are highly specific DNA cleaving enzymes, found within all forms of microbial life as well as in eukaryotic mitochondria and chloroplasts, that are encoded by genes that display genetic mobility and persistence. The activity of these proteins is directly responsible for the genetic behavior of their corresponding reading frames, by inducing homology-driven gene conversion events at the site of the DNA double-strand break that result in invasion by the endonuclease gene. When the homing endonuclease gene is embedded within a self-splicing element (a microbial intron or intein), the homing endonuclease gene is further enabled with the ability to invade coding sequences within their hosts’ genomes. Studies of the genetic behavior of homing endonuclease genes and of the structure and function of their endonuclease gene products over the past several decades have provided enormous detail on their evolution and function, and have allowed several types of homing endonucleases to be engineered and used for applications that require targeted gene modification.

**Figure 1:**
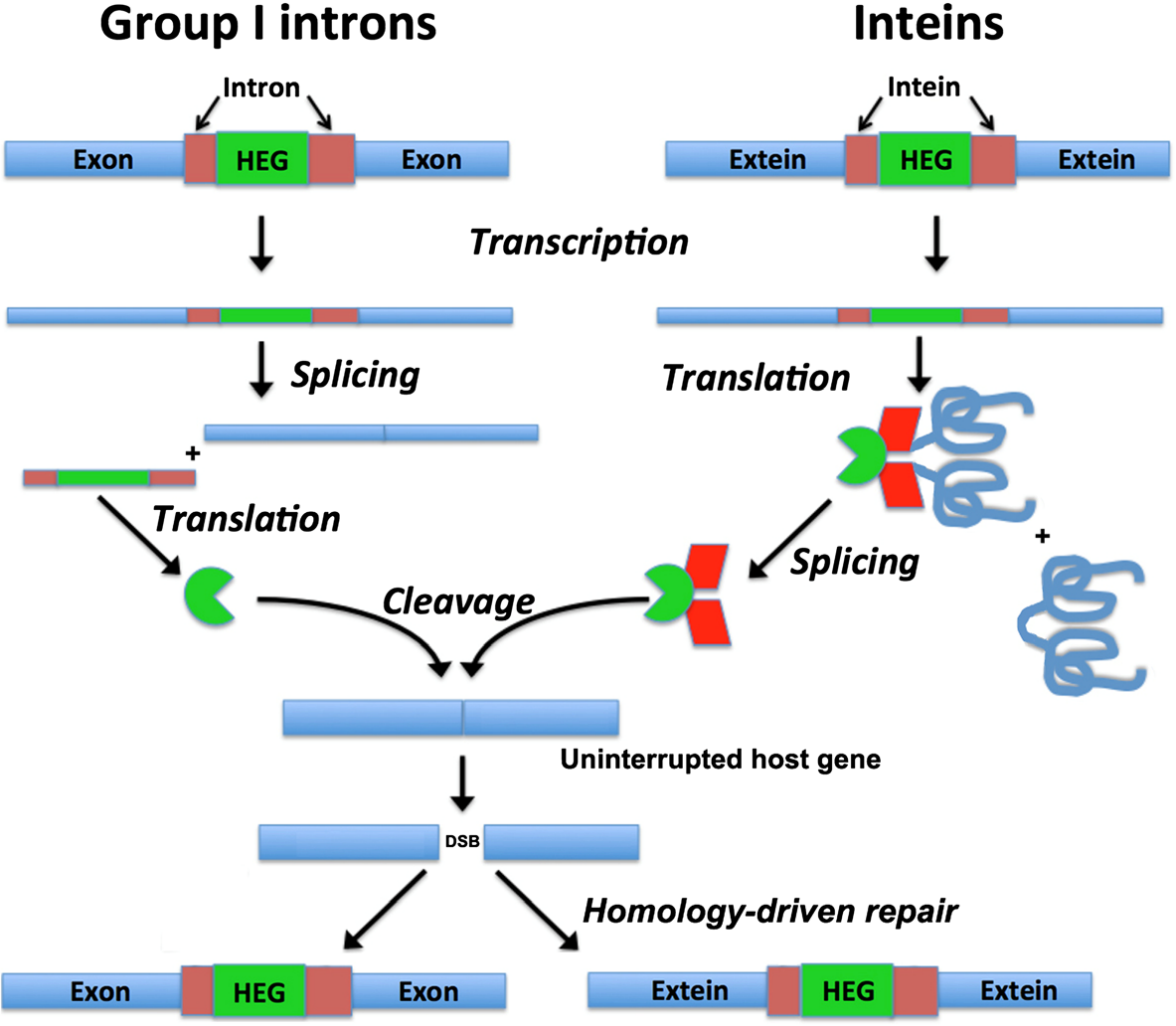
**Generalized homing mechanisms for mobile group I introns and inteins.** In both cases, the activity of the endonuclease (which is translated either as a free-standing protein from the intron, or as a fusion with the surrounding intein) leads to a double-strand break in an allele of the host gene that does not contain the intervening sequence. Subsequent repair via homology-driven strand invasion and recombination and DNA replication, using the allele containing the intron or intein (as well as the associated endonuclease coding sequence), completes the homing process. HEG, homing endonuclease gene.

The discovery of mobile introns and their homing endonucleases dates back to the 1970s. In 1978, an intervening sequence within a yeast mitochondrial ribosomal DNA (rDNA) was visualized using electron microscopy [1]. A subsequent study [2] described the sequence and organization of this yeast element, concluding that the rDNA was interrupted by an insertion of approximately 1 kb. Taken together, these papers provided the initial details corresponding to a locus in the yeast mitochondrial genome, termed ‘omega’, that had previously been observed to display dominant, non-Mendelian inheritance in mating experiments, a phenomena that eventually became known as ‘homing’ (Figure 1) [3].

Within 2 years, the complete nucleotide sequence of that mobile element, corresponding to a group I intron, was determined from several yeast strains. These analyses indicated that the intron was exceptionally long (1,143 base pairs), and contained an apparent reading frame that might encode a 235 residue protein [4]. A comparison of rDNAs from divergent organisms demonstrated that introns with similar organizations appeared to exist at a variety of positions within that otherwise highly conserved host gene, leading to a suggestion that these elements were recent additions to their mitochondrial genomes.

A similar study of intron sequences in the yeast mitochondrial cytochrome b (*cob*) gene, reported in the same year, demonstrated the presence of a different intron-encoded protein that appeared to be responsible for “mRNA maturase” activity (a function required for splicing and maturation of the cytochrome b message) [5]. Thus, the presence and sequence of several intron-encoded protein factors, and hypotheses describing two different biological functions (intron homing and intron splicing) were established in the literature within a period of several months.

Shortly after the initial descriptions of these intron-encoded reading frames, studies of a seemingly unrelated biological phenomenon provided the first hints of a biochemical mechanism that would eventually be linked to the process of intron homing [6,7]. Termed the ‘yeast mating type switch’, this process required the action of a site-specific endonuclease (at the time, termed ‘YZ endonuclease’) that was found to drive the homology-driven conversion of the yeast mating type (MAT) locus. A gene at that site encodes a transcription factor which activates either of two different suites of genes that control MAT: the DNA cleavage event driven by this endonuclease-induced recombination between MAT and a ‘hidden MAT’ locus. In subsequent years, the YZ endonuclease was renamed the HO endonuclease, and found to belong to the LAGLIDADG protein family. The observations in these early studies, which pre-dated the first biochemical characterization of a LAGLIDADG homing endonuclease, reported many of the eventual hallmarks of their properties, including the description of a long DNA target site and the observation of variable fidelity at several base-pair positions within that site. The actual notation of the conserved LAGLIDADG protein motif, which is found in many proteins involved in intron mobility, intron splicing and MAT gene conversion, was subsequently facilitated by the identification and sequencing of a sufficient number of intron-encoded proteins to allow its identification [8].

In 1985, several research groups demonstrated that translation of an intron-encoded protein, from the large rDNA gene in *Saccharomyces cerevisiae* mitochondria, was required and responsible for that intron’s mobility and inheritance, and that a double-strand break formed *in vivo* either at or near the site of the intron insertion was required for that process [9-11]. While these investigators noted that this intron behavior was somewhat similar to transposase function, they further indicated that the homing process appeared to correspond to a non-reciprocal recombination event at the cleavage and intron insertion site (that is, at ‘omega’), and was accompanied by co-conversion of DNA sequence tracts flanking the intron. Subsequently, the catalytic activity and specificity of the protein, and its probable role in creating a double-strand break at *omega*, was demonstrated using purified recombinant protein [12]. Subsequent analyses firmly established that the protein appeared to recognize a target site spanning approximately 20 base pairs in length, and demonstrated that the protein displayed a complex pattern of recognition fidelity across that target site [13].

While many of the seminal observations regarding homing endonuclease function were made using genetic information and systems derived from fungal mitochondria, additional studies on similar mobile elements in algal chloroplasts further demonstrated their ubiquitous distribution and the generality of their ability to invade host genes. In particular, studies of the I-CreI homing endonuclease from *Chlamydomonas reinhardtii* further established the roles played by the enzyme and a surrounding intron in genetic mobility and persistence, as well as reinforcing the concept that flanking homology regions near the site of an enzyme-induced double-strand break are critical for gene conversion [14-16]. Subsequent analyses of the distribution inheritance of additional mobile introns and homing endonucleases derived from algal chloroplasts, such as I-CeuI and I-MsoI, demonstrated that organellar genomes (and in particular, their rDNA genes) are often densely populated with such mobile elements [17-19].

In 1990, an examination of an unusual gene structure encoding a yeast vacuolar ATPase led to the discovery of a novel form of splicing, in which the intervening sequence was translated in-frame with the surrounding host gene, and then precisely excised (without the aid of auxiliary factors) post-translationally [20]. Found within that element, which was eventually termed an ‘intein’, was a sequence that again harbored sequence similarity to the LAGLIDADG protein family. Similar to the role of intron-encoded endonucleases in homing, the resulting protein product (an in-frame fusion of endonuclease and surrounding intein) was found to be responsible for the mobility and invasiveness of the entire intervening sequence [21]. The corresponding protein construct (eventually named ‘PI-SceI’) eventually served as one of the first homing endonucleases to be characterized mechanistically [22,23].

While much of the basic molecular biology of mobile introns and their homing was established by studies of intervening sequences isolated from organellar genomes in fungal and algal hosts, a series of subsequent studies using phage-derived mobile introns were critical for firmly establishing several additional details of that process. Shortly after the discovery of introns within phage genomes [24], investigators determined that many of them display mobility that is the result of intron-encoded homing endonucleases [25]. The ability to conduct quantitative homing assays using phage, both as a gene delivery vehicle and as a genetic recipient for mobile introns, allowed investigators to systematically characterize the mechanism and efficiency of intron transfer events to recipient alleles. These experiments demonstrated: (i) that homing is associated with co-conversion of flanking sequences that reflect the recombination process involved in the process of intron mobility [26]; and (ii) that the homing event does not specifically require the actual presence of an intron or intein, but instead is dependent only upon the expression of the endonuclease, the presence of its target site in acceptor DNA, the presence of sufficient homology between the DNA acceptor and donor, and the availability of phage- or host-encoded recombinase and exonuclease activities [27]. Subsequent studies demonstrated that intron mobility occurs in the context of phage recombination-dependent replication, and that homology-driven intron transfer can occur via multiple competing strand invasion pathways [28].

## Review

### Structures, functions and mechanisms

The experiments described above provided the initial examples of mobile group I introns and their corresponding homing endonucleases. Subsequent studies extending through the late 1990s demonstrated that similar mobile elements, each driven by intron-encoded proteins, are encoded across a vast array of organellar genomes, microbial genomes (including eubacteria, archaea, fungi, algae, and protists), and phage (see [29] for a review written during that time, and [30] for an additional review written this year). The transfer, duplication and transmission of these sequences was shown to be extremely efficient, leading to unidirectional gene conversion events in diploid genomes [9], possible horizontal transfer between phage and eukarya [31,32], competition between mobile introns in mixed phage infections [33], movement of introns between different subcellular compartments in unrelated organisms [34] and the rapid spread of mobile introns into related target sites throughout a broad range of biological hosts [35]. Although homing endonucleases can also be encoded by free-standing reading frames, their association with self-splicing sequences frees them to invade highly conserved sequences in protein- and RNA-encoding host genes, and then to persist in microbial genomes that are otherwise subject to selective pressure to eliminate extraneous genetic elements [36]. The sheer number and density of homing endonucleases and associated introns found to occupy various genomes and host genes can be extremely high. For example, the genome of T4 phage is found to contain 12 free-standing and 3 intron-encoded homing endonuclease genes (encompassing 11% of the total coding sequence in that phage’s genome) (reviewed in [37]).

**Figure 2:**
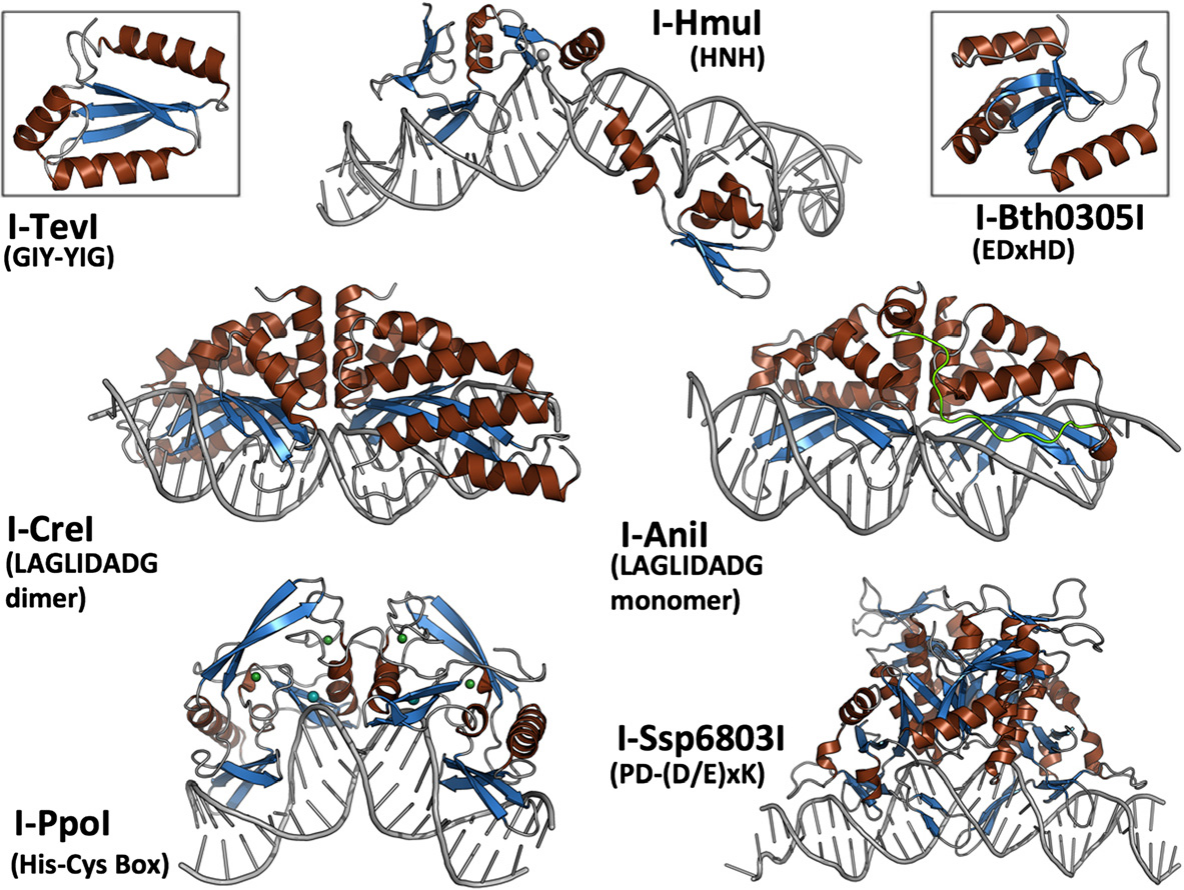
**Representative structures of homing endonuclease families and subfamilies. Top**: three separate types of catalytic nuclease domains (GIY-YIG, HNH and EDxHD) are found in various phage-encoded homing endonucleases (as well as less commonly in organellar genomes). As illustrated by the structure of full-length HNH endonuclease I-HmuI (middle), these nuclease domains are coupled to elongated DNA-binding regions that are involved in contacts to long target sites. Shown in the insets are crystal structures of the catalytic domains of the GIY-YIG endonuclease I-TevI (left) and the EDxHD endonuclease I-Bth0305I (right). Both of those endonucleases display a general domain organization that is similar to that of I-HmuI: a nuclease cleavage domain tethered to an extended DNA binding region that contains multiple structural motifs. **Middle**: two closely related types of LAGLIDADG homing endonucleases, corresponding to homodimeric and monomeric enzymes, are encoded within organellar and archaeal genomes. Whereas the homodimeric enzymes can be applied to genome engineering after converting their quaternary organization into an artificial monomeric protein (by tethering the two equivalent domains to one another with a peptide linker) the wild-type monomeric enzymes can be used directly for that purpose. In either case, the N- and C-terminal domains of the protein can be individual engineered and then fused to create highly specific gene targeting proteins. **Bottom**: His-Cys box endonucleases (which harbor a variant of the HNH active site) and PD-(D/E)xK endonucleases are found in protist and cyanobacterial genomes. Both enzymes are multimers (a homodimer and a tetramer, respectively).

At least six unique families of group I homing endonucleases (‘LAGLIDADG’, ‘HNH’, ‘His-Cys box’, ‘GIY-YIG’, ‘PD-(D/E)xK’ and ‘EDxHD’ proteins) have been described over the past 25 years (reviewed in [38]). Each is named based on the presence of conserved sequence motifs that correspond to conserved structural and catalytic residues in each family’s catalytic domain and active site, and each is largely (although not absolutely) confined to a well-defined host range. Structural analyses of members from each of these families (Figure 2) demonstrate that they have embedded their nuclease catalytic cores in a wide variety of surrounding protein scaffolds, and appear to be descended from multiple, unique ancestral nucleases.

A hallmark of all homing endonucleases, regardless of their family origin, is the contrast between their small size (homing endonuclease genes usually encode proteins that comprise fewer than 200 residues) and their long DNA target sites (which often extend to well over 20 base pairs). The determination of the first structures of representatives from each of these homing endonuclease families [39-50] illustrated two strategies that allow such compact proteins to bind long DNA sequences. The majority of homing endonucleases that are most commonly derived from phage (that contain either an HNH [46], a GIY-YIG [49] or an ‘EDxHD’ [47] catalytic domain) form highly elongated proteins with minimal hydrophobic cores. They rely upon the presence of additional DNA binding regions (often termed Nuclease-associated modular DNA-binding domains or ‘NUMODs’ [51]) that are loosely tethered to their catalytic domains, and thereby contact DNA target sites up to 30 base pairs in length. At least one of these phage-derived homing endonuclease families (the most recently described ‘EDxHD’ enzymes, exemplified by the I-Bth0305I endonuclease) appears to employ both of these strategies, by forming a long multi-domain structure while also dimerizing on an exceptionally long DNA target that extends to nearly 60 base pairs in length [47].

In contrast, many homing endonucleases found in archaea, eukarya and eubacteria (corresponding to the LADLIDADG [43], His-Cys box [41] and PD-(D/E)xK [50] proteins) display more compact protein folds that usually multimerize and thereby double their DNA-contact surface. This strategy constrains the endonuclease to recognition of a DNA sequence that contains significant palindromic symmetry. Only one subfamily of homing endonucleases (the monomeric LAGLIDADG enzymes) display compact, globular protein folds and also recognize completely asymmetric DNA target sites [39,45]. Those enzymes are among the largest of homing endonucleases, often containing nearly 300 residues that are distributed across two pseudo-symmetric protein domains.

### DNA recognition

Analyses of insertion sites for mobile group I introns and the corresponding cleavage sites for their homing endonucleases indicate that they are often found at positions and sequences within their host genes corresponding to coding sequences that span critical residues within an enzyme active site, a ligand-binding pocket, or a strongly conserved structural motif. In one particularly notable example, an exceptionally well-conserved sequence in a large rDNA, that encodes a structural helix at the ribosomal RNA interface and is located near a transfer RNA (‘tRNA’) binding site and the peptidyl transferase active site, has been independently invaded three times: in protists by introns armed with His-Cys box homing endonuclease genes [52], in archaea by introns armed with LAGLIDADG homing endonuclease genes [53] and in metazoans by a retrotransposon [54]. Thus, a sequence that is sufficiently invariant over the course of evolution can become a repeated target for invasion by mobile elements, including homing endonucleases.

DNA recognition mechanisms vary widely across the families of homing endonucleases described above, but in each case these mechanisms strike a balance between the somewhat orthogonal requirements of (i) recognizing a target of sufficient length to avoid overt toxicity in the host, while (ii) accommodating at least a small amount of sequence drift within that target. The LAGLIDADG and His-Cys box enzymes, which are the most sequence-specific of these proteins, rely upon extensive, antiparallel DNA-binding β-sheets that dock into the major grooves of their target sites [41,43,55]. Nearly one-quarter of the amino acids in the entire endonuclease participate in the resulting protein-DNA contacts. There they establish a collection of sequence-specific and non-specific contacts that comprise many directional hydrogen bonds to individual bases, water-mediated contacts, and additional steric contacts that further enforce specificity. These contacts are distributed non-uniformly across base pairs throughout the target site. DNA bending near the middle of each target appears to further contribute to sequence-specificity.

In contrast, the less specific homing endonucleases, found primarily in phage, often form a more heterogeneous collection of DNA contacts within the major and minor groove, as well as across the backbone, of their target sites. These enzymes (typified by I-TevI (a GIY-YIG endonuclease) [49], I-HmuI (an HNH endonuclease) [46] and I-Bth0305I (an ED-HD endonuclease) [47]) all display extended, multi-domain protein structures in which disparate structural elements that include individual α-helices, zinc fingers and/or helix-turn-helix domains. These regions of the proteins contact DNA targets that can span up to 30 base pairs. Although the overall specificity of these endonucleases is considerably lower than their eukaryotic and archaeal cousins, these endonucleases also can display elevated specificity at base pair positions within the target that are strongly constrained in the host gene [47,56-58].

The specificity profiles and overall frequency of DNA cleavage exhibited by homing endonucleases has been particularly well characterized for the LAGLIDADG family, which comprise the most specific of the homing endonucleases and are most commonly used for applications in gene targeting and genome engineering. Studies of the target sites and specificities of three of the earliest identified examples of these enzymes (the monomeric I-SceI endonuclease, the homodimeric I-CreI, and the intein-encoded PI-SceI) each indicated that the overall length of their target sites was 20 base pairs or greater. Their fidelity of recognition, as evaluated by the effect of base-pair substitutions within the target on cleavage activity, was highly variable across the target site [59-61]. Subsequent comparison of the I-CreI specificity profile with the distribution of atomic contacts throughout the protein-DNA interface indicated that specificity was largely derived by a large number of direct and water-mediated contacts between protein side chains and nucleotide bases, particularly across a series of at least 3 consecutive base pairs in each half-site [55]. In addition, the bending and distortion of the DNA target near the center of the site appears to elevate total target specificity and contribute heavily to cleavage fidelity at the four ‘central’ base pairs that reside between the scissile phosphates on each DNA strand (LAGLIDADG enzymes always generate a pair of 4-base, 3’ overhangs).

Subsequently, a series of much more detailed analyses of the specificity profile of a single LAGLIDADG enzyme (I-AniI) were conducted, utilizing three separate, complementary approaches: i) an *in vitro* selection experiment for cleavable substrates, extracted and amplified from a library of randomized target site variants [62]; ii) direct examination of relative binding affinity and cleavage activity for the enzyme against all single base pair variants of the enzyme’s target site using surface-display and flow-assisted cell sorting (FACS) analyses of metal-dependent binding and cleavage [63]; and iii) direct examination of relative binding affinity and cleavage activity against all single base pair variants of the enzyme’s target site, using purified enzyme and corresponding biochemical measurement of relative cleavage rates and binding affinities [64].

These experiments, in addition to thoroughly characterizing the specificity of one particular homing endonuclease, provided considerable insight into the behavior of LAGLIDADG enzymes. Collectively, the experiments indicated that:

1) The overall specificity of the enzyme, in terms of expected cleavage frequency versus random DNA target sequences, is approximately 1 in 10^8^ (and possibly somewhat more specific, because the extent to which base pair substitutions, that are individually tolerated by the enzyme, would be accommodated simultaneously is unclear).

2) The positions within the target site where base pair substitutions are particularly well-tolerated by the enzyme, corresponding to ‘promiscuous’ recognition, are well-correlated with loosely constrained ‘wobble’ positions in the coding frame of the underlying host gene (the mitochondrial cytochrome B oxidase gene in *Aspergillus nidulans*).

3) Many substitutions in the target site that cause decreased cleavage activity often do so primarily via a reduction in substrate binding affinity or through a reduction in substrate cleavage rate. In the case of I-AniI, these two different effects map rather cleanly to the two DNA-half sites, and appear to reflect an inherent asymmetry in the role of each protein domain (and the corresponding DNA half-sites) in target site binding and cleavage.

Additional data on the *in vivo* specificity of homing endonucleases, and whether their activity profiles differ significantly from those measured using *in vitro* methods, are relatively scarce. However, at least one recent analysis of the apparent cleavage targets of I-SceI in transfected human cells [65] has indicated that, for at least one LAGLIDADG enzyme, a significant number of chromosomal target sites (including some that differ significantly from the canonical *sce* target sequence) appear to be cleaved.

### DNA cleavage

**Figure 3:**
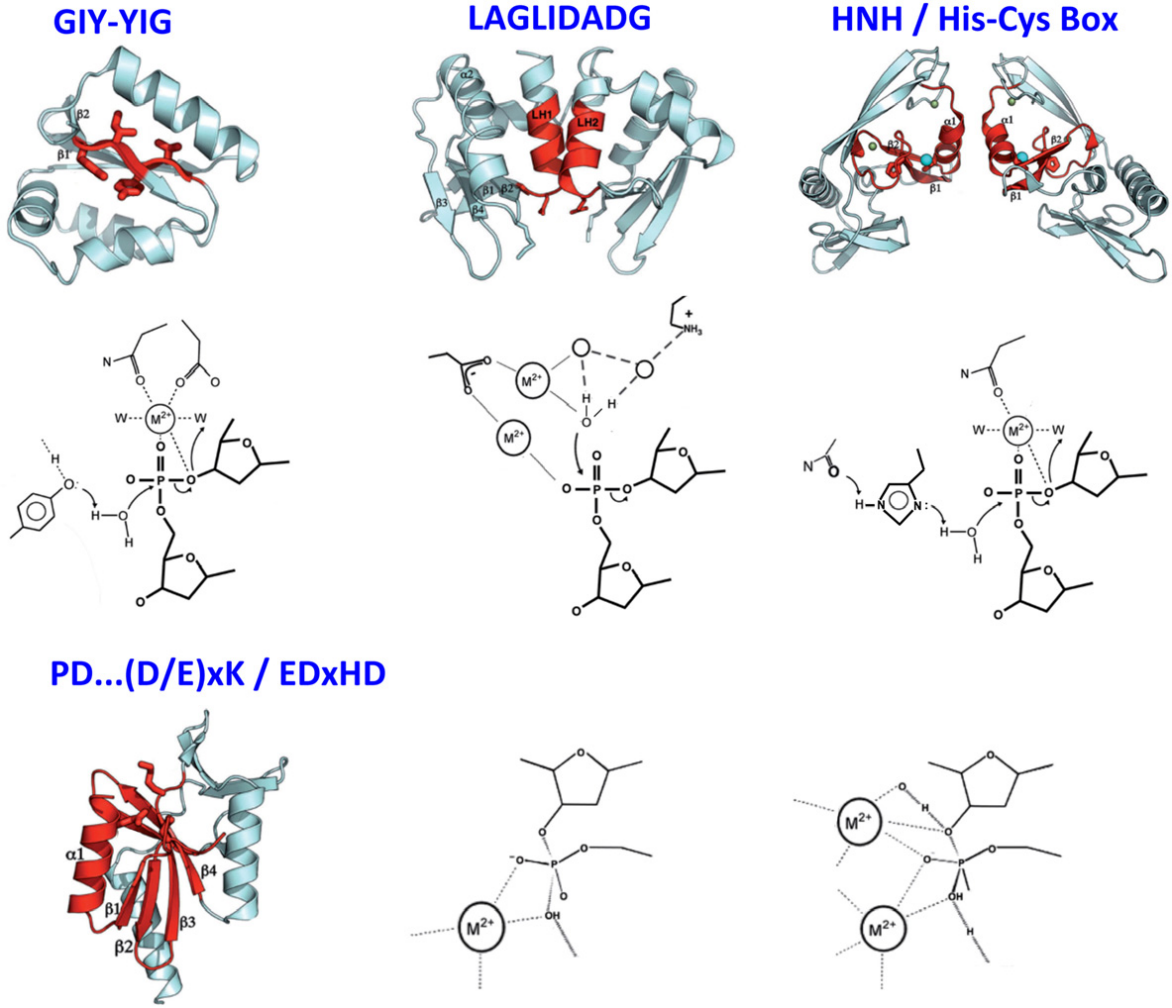
**Representative active sites and generic mechanisms of DNA cleavage by homing endonuclease families.** The HNH and His/Cys box endonucleases contain similar nuclease motifs and active sites, and are thought to be related via divergence from a common ancestor. In those enzyme families, an absolutely conserved active site histidine residue directly deprotonates a water molecule; the ability of the histidine side chain to act as a general base is facilitated by a hydrogen bond to a neighboring carbonyl moiety (usually an asparagine side chain). The GIY-YIG endonucleases use a similar mechanism, with the difference that an active site tyrosine appears to serve a similar role as an activated general base, again to deprotonate the incoming nucleophilic water molecule. In contrast, the PD-(D/E)xK and EDxHD endonucleases display similar active site structural motifs and mechanisms that appear to be similar to previously well-characterized type II restriction endonucleases; in those enzymes a metal-bound water molecule acts as the incoming nucleophile. In these enzymes (corresponding to either the restriction or the homing endonuclease catalysts) the precise number of metal ions employed is often not entirely clear (and hence is represented in the figure either as a single-metal or a two-metal-dependent active site). In each panel of the figure, the most conserved catalytic elements (corresponding to those regions that contain the enzymes’ namesake motifs) are shown in red, and the corresponding secondary structural elements of the catalytic cores are labeled. LH1 and LH2 in the middle panel refer to LAGLIDADG helices 1 and 2 in a monomeric LAGLIDADG homing endonuclease.

Many of the mechanisms and corresponding active site architectures by which a phosphodiester bond can be hydrolyzed [66] are observed for the various families of homing endonucleases (Figure 3). For all of these enzymes, the reaction proceeds according to a metal-dependent hydrolysis reaction, without the formation or accumulation of a covalent enzyme-DNA intermediate. Biochemical and structural analyses indicate that they all utilize an activated water molecule as the incoming nucleophile, which drives an in-line S_N_2 displacement of the 3’ leaving group, resulting in the formation of 5’ phosphate and 3’ hydroxyl product ends. They utilize either a strong general base to deprotonate the incoming water molecule, and/or a bound metal ion to significantly decrease the pKa of the water molecule, as well as an appropriate electropositive group positioned to stabilize the phosphoanion transition state and a proton donor to neutralize the 3’ hydroxylate leaving group.

Different homing endonuclease families display different strategies by which these basic mechanistic requirements of a nuclease reaction are satisfied (Figure 3). The LAGLIDADG, PD-(D/E)xK and EDxHD nucleases all appear to utilize variations of a traditional two-metal hydrolysis reaction, in which a metal-bound hydroxyl serves as a nucleophile, and a second bound metal ion is appropriately positioned to stabilize the phosphoanion and the 3’ leaving group [47,67,68]. The LAGLIDADG active site is particularly unique in that: (i) the nucleophilic, metal-bound water is surrounded solely by a network of additional ordered solvent molecules, rather than being directly coordinated by protein side chains; and (ii) the two active sites (which are closely juxtaposed in order to cleave phosphates on either side of the DNA minor groove) often appear to share a common metal ion [69].

In contrast, the HNH, His-Cys Box and GIY-YIG endonucleases all appear to follow a reaction mechanism in which the incoming water molecule is not associated with a bound metal ion, but instead is in direct contact either with the side chain of a conserved histidine or tyrosine residue [46,48,70]. In either case, the activation of the nucleophilic water molecule require a strong enough general base to abstract a proton from a water molecule that is not associated with nearby metal ion.

### Additional and related functions

The most obvious biological function of a homing endonuclease is to drive the mobility, invasiveness, and persistence of its own coding sequence; as such the protein is the product of a specific form of ‘selfish DNA’. However, this function is largely independent of host-derived selection pressure, because the mere presence and persistence of a mobile intron does not provide any obvious benefit to the host. As a result, mobile introns and their associated homing endonuclease genes are observed to undergo a relatively rapid evolutionary cycle of invasion, mutational degradation of its form and function, and precise deletion from the host genome (which produces a site that is once again subject to invasion) [71].

Perhaps as a way to increase selection pressure for maintenance of a stable protein fold after gene invasion, some homing endonucleases have acquired an additional biological function that may provide a benefit to the host. The most well-documented of these functions, termed ‘maturase’ activity, corresponds to the direct interaction of the intron-encoded protein with the surrounding intron - a specific, high-affinity binding activity that is required to ‘chaperone’ the RNA element through required steps of folding that lead to its eventual splicing [5]. In some cases, closely related intron-encoded proteins may each display only a single activity (that of either an endonuclease or a maturase) [72]; in at least one case only a single amino acid substitution in a monofunctional maturase was needed to restore endonuclease activity [73]. In other cases, an intron-encoded protein may display bifunctionality, acting both as an active endonuclease and as a maturase. In the most well studied example of such a dual-function protein (the I-AniI endonuclease/maturase) the surfaces and residues of the protein involved in DNA and RNA recognition were found to be different, and the two activities could be uncoupled through separate point mutations that disrupted each activity [39,74].

Another system in which a homing endonuclease displays a secondary function with potential benefit to the host is the T4 phage-encoded I-TevI endonuclease, which displays not only DNA cleavage activity, but also acts as a transcriptional autorepressor of its own expression [75]. This secondary activity depends upon the endonuclease binding a DNA sequence that overlaps a late promoter within the 5’ region of its own reading frame - a function that is essential for optimal splicing activity of the surrounding intron, in order to avoid compromising the expression of the host gene. The *cis* regulatory sequence that is recognized by the DNA binding domain of I-TevI is similar, but not identical, to comparable base-pair positions in the enzyme’s cleavage target. However, the absence of an appropriately positioned upstream consensus sequence (5’-CNNNG-3’) for recognition by the nuclease domain greatly reduces the cleavage activity of the enzyme at the regulatory site, allowing the I-TevI protein to function as a transcriptional repressor.

Beyond the acquisition of secondary functions and activities by active homing endonucleases, there are clear evolutionary connections between these proteins and a wide variety of host proteins and functions (recently reviewed in [76]). Proteins that share common folds and catalytic motifs with homing endonucleases are found in proteins that participate in phage restriction, in DNA repair, in processing DNA junctions and cross-over structures during strand invasion events that lead to integration, transposition and recombination, in transcriptional regulation, in chromatin remodeling and maintenance, and in gene conversion events. While the relative origin(s) and sequence of events that led to the establishment of modern day homing endonucleases and related host proteins is not obvious, it seems clear that a small number of ancient DNA binding nucleases have served as common ancestors for a wide variety of proteins that are engaged in competing functions of genomic invasion and genomic fidelity.

### Application for genome engineering

Genome engineering and targeted gene modification is a rapidly maturing discipline in which genomes within cell lines, tissues or organisms are manipulated and altered at specified individual loci [77]. The first demonstrations that the introduction of a site-specific nuclease into a mammalian genome could increase the efficiency of a site-specific sequence conversion event were conducted using the I-SceI LAGLIDADG endonuclease [78-80]. In those studies, the wild-type target site of I-SceI was first introduced into a desired chromosomal allele, prior to the subsequent introduction of the endonuclease. While this strategy did little to simplify the process of targeted gene modification, it demonstrated that highly specific endonucleases that generated double-strand breaks at unique loci in complex eukaryotic genomes could greatly enhance the efficiency of corresponding gene modification processes at those positions. Within 2 years of those studies, the first artificial zinc-finger nucleases (ZFNs) had been described [81], and the race to develop and apply them for specific genome editing purposes had begun.

Four separate macromolecular scaffolds, which each generate site-specific double-stranded DNA breaks, can now be used for targeted gene modification: ZFNs (first described as genome editing tools in [82,83]); transcriptional activator like (TAL) effector nucleases (TALENs) [84]; the clustered regularly interspaced short palindromic repeats (CRISPR)-Cas9 (‘CRISPR’) system [85-87]; and LAGLIDADG homing endonucleases (now also termed ‘Meganucleases’) [88]. Thus, the field of site-specific genome engineering using site-specific nucleases enjoys a wealth of molecular scaffolds. Three are protein based and one relies on RNA-guided specificity for gene targeting.

**Figure 4:**
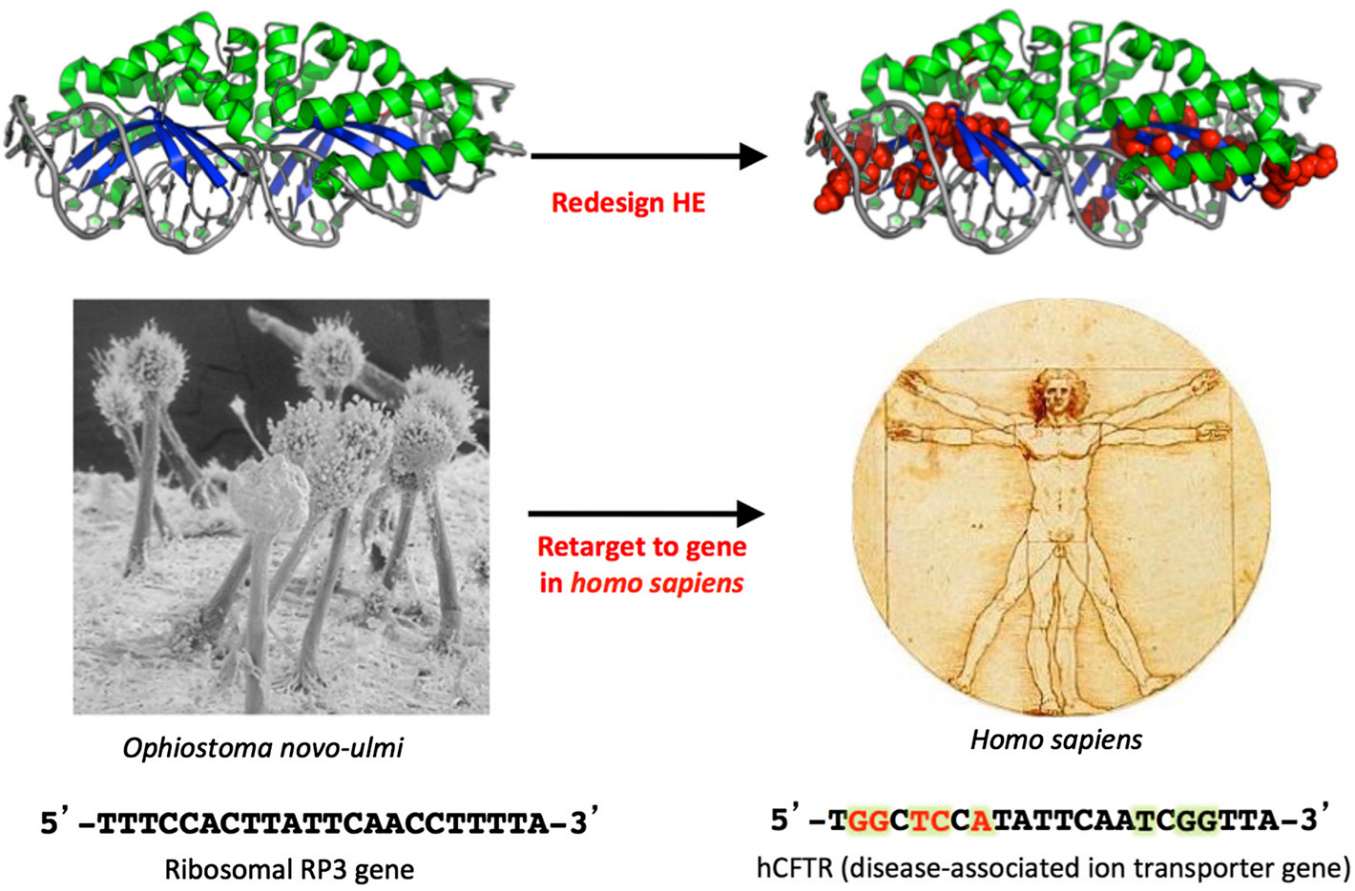
Redesign of a LAGLIDADG homing endonuclease (HE; also termed a ‘meganuclease’) for a specific genome engineering application (such as modification of a disease-associated human gene locus) involves the alteration of a substantial fraction of its DNA-contacting residues, as well as further optimization of neighboring positions on the protein scaffold.

The ease of constructing CRISPR-based gene targeting nucleases (and, to almost the same extent, of constructing TAL nucleases) has led to an explosion of activity in the field of nuclease-induced targeted gene modification experiments, and corresponding excitement concerning the potential of targeted genome engineering [89]. In contrast, the utility of LAGLIDADG homing endonucleases has been somewhat dismissed, on the assumption that the ‘degree of difficulty’ for retargeting their recognition profiles for a desired genomic target is too high (because their DNA recognition mechanisms cannot be reduced a simple modular ‘code’) (Figure 4). For the construction of genetically altered (‘transgenic’) model organisms and corresponding cell lines for research, this outlook is mostly appropriate. However, for therapeutic applications, which demand the highest level of targeting specificity, combined with high levels of gene modification activity, the continued development of compact, highly specific nuclease domains as an alternative to nonspecific nuclease domains that rely upon additional DNA targeting moieties seems appropriate. A recent proof of principle has demonstrated the possibility of replacing the R.FokI nuclease domain with the catalytic domain of the I-TevI homing endonuclease for the purpose of creating a site-specific, single chain nuclease with elevated specificity at the actual point of DNA cleavage [90], as well as experiments that have fused the more specific R.PvuII nuclease domain to TAL effector or zinc finger DNA binding domains [91,92].

Soon after the original ‘proof of concept’ studies with I-SceI [78-80], it became obvious that modification of a homing endonuclease’s cleavage specificity would be required in order to target and modify endogenous target sites in various biological genomes. The determination of the first DNA-bound structures of homing endonucleases (I-PpoI and I-CreI in 1998 [43,93] and then I-MsoI, I-AniI and I-SceI in 2003 [39,45,55]) allowed identification of the amino acids in each system that were found within contact distance of base pairs in their DNA targets, both individually and within distinct ‘clusters’. Armed with such information, a series of experiments of increasing complexity, all designed to alter the DNA cleavage specificity of homing endonucleases, were reported, eventually leading to the ability to completely retarget homing endonucleases for the modification of unique genomic targets.

#### (i) Alteration of homing endonuclease target specificity at individual base pairs

Early studies provided multiple examples where mutation of individual residues in a homing endonuclease DNA-binding surface resulted in a change in the specificity at a single position in the target site [60,94]. The earliest experiments to alter LAGLIDADG endonuclease specificity often relied upon *in vitro* or cellular assays to visually identify mutated endonuclease constructs that displayed altered recognition specificity. Some of these early protocols utilized reporters of high affinity DNA binding (for example, through the use of a bacterial two-hybrid screening strategy) [60] or methods that coupled endonuclease activity to the elimination of a reporter gene [94,95].

At the same time, an experiment that relied on structure-based redesign of the protein-DNA interface to alter specificity at a single base pair, relying upon computational algorithms that repack and optimize new protein-DNA contacts, was also reported [96]. In that study, the redesigned enzyme bound and cleaved a corresponding recognition site harboring a single base pair substitution 10^4^ times more effectively than did the wild-type enzyme, with a level of target discrimination comparable to the original endonuclease.

#### (ii) Combined alteration of specificity at multiple, adjacent base pairs

By 2004, it was apparent that, in some cases, alteration of individual DNA-containing side chains in homing endonucleases might result in desirable and useful changes in specificity at individual base pairs in the target [97]. However, it was not clear to what extent such alterations might be combined in ‘additive’ steps for a complete redesign process, to more significantly alter the protein’s DNA binding and cleavage specificity. As a way forward, a particularly powerful selection method to screen a homing endonuclease library for altered DNA cleavage specificity was described in 2005, in which the protein’s cleavage activity was coupled to the homology-driven reconstitution of a reporter gene [98]. This method was used to systematically screen multiple semi-randomized libraries of the I-CreI homing endonuclease, where each library harbored collections of amino acid substitutions within ‘modules’ or ‘clusters’ of residues that collectively contacted several adjacent DNA base pairs. By doing so, investigators could isolate and characterize a large number of individual protein variants, harboring multiple amino acid changes that could accommodate multiple adjacent base pair substitutions at several distinct regions of the enzyme’s target site [99,100].

Aside from building up a large collection of variants of the I-CreI enzyme that could cleave DNA target sites harboring many different clusters of altered base pair sequences, these studies also demonstrated that the output of such screens was more complex than might be predicted based on prior studies of changes to single amino acids in the protein-DNA interface. Alteration of individual protein side chains that caused reduced activity or specificity were sometimes well tolerated in more extensively altered pockets; conversely, some alterations of protein-DNA contacts that behaved well on their own were found to be incompatible with substitutions at adjacent positions (reviewed in [101]).

A separate effort, again conducted using structure-based computational redesign methods, to create a similar specificity change that involved multiple consecutive base pairs also recapitulated the results of the selection-based experiments [102]. The concerted redesign of the I-MsoI homing endonuclease to accommodate base pair substitutions at three consecutive positions was more successful than attempts to employ incremental or sequential redesign for recognition of individual substitutions, highlighting the importance of context-dependent optimization of protein-DNA interactions. Crystallographic structure analyses of all the redesigned enzymes in this study indicated that the basis of this behavior could be observed in patterns of structural context-dependence, extending across a local network of adjacent side chains and corresponding DNA base pairs, that caused unpredictable differences in DNA backbone conformation and side chains rotamers.

#### (iii) Domain shuffling

The concept that domain swaps between different wild-type homing endonucleases might be possible could greatly increase the number of such scaffolds for genome engineering (in theory, shuffling the N- and C-terminal domains of 10 wild-type endonucleases could yield up to 100 unique DNA-cleaving proteins with different target specificities). At the same time that the experiments described above were being performed, several studies demonstrated that entire domains or subunits from unrelated LAGLIDADG enzymes could be mixed and fused to create novel chimeric homing endonucleases that recognize corresponding chimeric DNA target sites [103-105]. These studies demonstrated that the individual domains and subunits of LAGLIDADG enzymes are largely responsible for the recognition and binding of individual DNA half-sites. Subsequent experiments reinforced this conclusion [103,106-108]. Most importantly, these studies demonstrated that the task of altering a homing endonuclease’s cleavage specificity could be ‘broken down’ into two separate redesign projects to individually target the left and right half-sites of a DNA target, by systematically altering the DNA-contacting residues of the protein’s N- and C-terminal domains and then combining the final solutions for each domain into a single gene targeting protein.

To further explore this concept, more recent studies focused on systematic exchange of domains between homing endonucleases selected from a relatively closely related clade (all from mesophilic fungal mitochondrial genomes, with 40 to 70% sequence identity between the individual proteins) [109]. Using a simple method in which limited variation was introduced into the domain interface, catalytically active enzymes were recoverable for approximately three-fourths of the resulting chimeras. While potentially useful for future creation of large numbers of gene targeting reagents, such domain fusions will probably prove to be largely unnecessary for genome engineering, because several research groups have demonstrated that such reagents can now be readily obtained starting from wild-type proteins, as described in the next section.

#### (iv) Complete retargeting of homing endonucleases and application to genome editing

Multiple groups (both academic and within the biotech industry) have recently exploited the data summarized above to generate and use completely retargeted and optimized homing endonucleases for genome engineering and targeted gene modification. The method employed by these groups can loosely be divided into strategies that either ‘go deep’ (by focusing on the maximum possible ‘redesignability’ of a single homing endonuclease) or that ‘go broad’ (by employing bioinformatics to choose from an increasing number of well-characterized wild-type endonucleases, followed by the redesign of the most appropriate starting scaffold for a given target). Both approaches have been shown to work, and in both cases the use of direct structure-based redesign and structure-based selection methods have each found their place as part of the engineering process. A survey of the recent literature demonstrates the increasing pace and speed at which highly active and extremely specific gene-targeting reagents can be generated from LAGLIDADG homing endonucleases.

Two separate biotechology companies, Cellectis Inc. (8 rue de la Croix Jarry 75013 Paris, France) and Precision Biosciences Inc. (302 East Pettigrew Street Durham, NC 27701 USA)) have each described the creation of extensively altered variants of the I-CreI homing endonuclease and their successful application for nuclease-driven, targeted gene modification. Because wild-type I-CreI is a natural homodimeric enzyme, both efforts rely upon the ‘monomerization’ of the I-CreI protein to create a single-chain reagent in which the two subunits of the enzyme are linked by a peptide tether and then expressed in *cis* as a monomeric scaffold [110-112]. Armed with this construct, redesign efforts can then be conducted on individual protein domains (targeting corresponding half-sites of the desired genomic target) with the resulting constructs combined into a single polypeptide which is further optimized for optimal *in vivo* performance. The strategies used to identify and combine individual amino-acid substitutions in the I-CreI scaffold differ between these two approaches. One group relies predominantly upon direct structure-based redesign of the wild-type protein [110], while the other relies upon the output of phenotypic screens from semi-randomized protein libraries [98]. Both approaches have largely converged on alteration of the same DNA-contacting protein side chains.

Using these approaches, these groups have created and employed redesigned variants of single-chain I-CreI endonuclease for a wide variety of purposes, such as modification and correction of the human *XPC* gene for the treatment of xeroderma pigmentosum [88,113,114], creation of cell lines harboring defined genetic insertions and alterations [115,116], generation of transgenic lines of maize containing heritable disruptions of the *ligueleless*-1 and *MS26* loci [110,117], excision of defined genomic regions in *Arabidopsis*[118], insertion of multiple trait genes in cotton [119], generation of *Rag1* gene knockouts in human cell lines [111,120] and in transgenic rodents [121], disruption of integrated viral genomic targets in human cell lines [122], and demonstration of the correction of exon deletions in the human *DMD* gene associated with Duchenne Muscular Dystrophy [123].

Yet another biotechnology company (Pregenen Inc. (454 N. 34th St. Seattle WA 98103 USA)) has employed both a different homing endonuclease scaffold (I-OnuI, which is a naturally occurring monomeric LAGLIDADG enzyme, rather than a ‘monomerized’ homodimeric protein) and a considerably different *in vitro* engineering pipeline that relies upon yeast surface-display and high-throughput flow cytometry to screen semi-randomized endonuclease libraries for altered binding and cleavage specificity [63]. Using this strategy, gene targeting nucleases have been created that drive the disruption of fertility-related genes as part of a gene drive strategy for the control of insect disease vectors [124], and that quantitatively disrupt the T-cell receptor α-chain gene (as part of a broader strategy to create engineered T-cells that can be used as anticancer immunotherapeutic reagents) [125]. Unlike the engineering strategies employed for I-CreI, which both rely upon relatively low-throughput screening of enzyme variants and/or minimally complex libraries that are reliant upon prediction of specificity-changing amino acid substitutions at direct contact points between protein and DNA, the platform used with I-OnuI relies upon the elevated throughput that can be realized through the use of yeast (a naturally recombinant host that facilitates creation of higher complexity libraries) and the speed of FACS screens.

More recently, an academic laboratory has described a complementary strategy for the purpose of retargeting of meganuclease specificity. Well-characterized wild-type meganucleases are computationally screened to identify the best candidate protein to target a genomic region; that endonuclease is then redesigned via activity selections within compartmentalized aqueous droplets [126]. The use of this system allows the formation and interrogation of exceptionally large libraries of randomized endonuclease sequences (sampling up to 10^10^ constructs per selection step) as well as the tight control of temperature, time and concentration during individual selection steps over the course of endonuclease engineering. In this study, the method was illustrated by engineering several different meganucleases to cleave multiple human genomic sites, as well as variants that discriminate between single nucleotide polymorphism (SNP) variants. Simultaneous expression of two such fusion enzymes results in efficient excision of a defined genomic region (a property that, combined with the small size and coding sequences of homing endonucleases, is particularly useful for such applications).

#### (v) Refinement and extension of engineered homing endonuclease technologies

Beyond the development and demonstration of reliable methods for engineering homing endonuclease, their use as gene targeting reagents has been further facilitated by several recent developments. First, the number of wild-type homing endonucleases that have been identified and characterized has grown rapidly, along with the cataloguing and public deposition of their most important features of protein sequence, target sites, and structural features of recognition [127,128]. Second, their unique ability to generate defined 3’ overhangs can enhance the recombinogenicity of their cleavage products, and also can be exploited for enhanced gene disruption through the parallel introduction of 3’ exonucleases [129,130]. Third, their active sites are amenable to the introduction of individual point mutations, with the goal of generating site-specific nickase enzymes that can be used to control the outcome of competing repair pathways [131,132]. Their compact size and the availability of free N- and C-termini has facilitated their fusion with auxiliary DNA targeting domains (in particular, through the addition of engineered TAL effector repeats) [125,126] to create highly specific and active gene targeting nucleases that still comprise small, single chain, easily packaged scaffolds. Finally, extremely informative reporter systems and assays that allow precise measurements and quantitation of the mechanisms, efficiency, and repair pathway choice and outcome(s) resulting from nuclease-induced double-strand breaks have been developed [133-135], facilitating the refinement and optimization of such systems for genome engineering applications.

## Conclusions

There is much to be learned from the history of studies of mobile introns and their associated protein factors (which has the advantage, from the point of view of the investigator tasked with writing this review, of starting with an obvious ‘big-bang’ moment corresponding to their initial discovery in 1971). From a biological standpoint, perhaps the most important insights are those gained by reducing the complexities that surround the co-evolution of a host and a parasitic endosymbiont down to the simplest level: that of a DNA binding protein tasked with the recognition of an evolving genomic target. This simple molecular drama, conducted over many generations and replete with many nuances and subtleties, continues to play out in every biological kingdom, using all known types of homing endonucleases, split gene structures, and host genes. At the same time, the functional capabilities of these small mobile elements are continually being spun-off into new and different biological pathways and functions, ranging from the protection and maintenance of the genome (an ironic twist given that the fundamental purpose of a homing endonuclease is to act as an invasive element) to the transcriptional regulation of complex developmental processes.

Beyond these scientific points, the importance of homing endonucleases for genome engineering speaks clearly of the impact, often unexpected and unpredictable, that basic research, even of the most seemingly esoteric or mundane type, can have on the creation of entirely new areas of biotechnology and medicine. Just as studies of bacterial phage restriction in the early 1950s led to the discovery and application of restriction endonucleases (molecules that, when harnessed, paved the way for the creation and use of recombinant DNA and the establishment of the biotechnology industry), the study of seemingly minor and unimportant genetic markers in yeast and phage provided the initial steps into a field of targeted genetic modification and genome engineering that may revolutionize much of the way in which future biological studies are conducted.

## Abbreviations

CRISPR: clustered regularly interspaced short palindromic repeats; FACS: flow-assisted cell sorting; MAT: mating type; rDNA: ribosomal DNA; TAL(EN): transcriptional activator like (effector nuclease); ZFN: zinc-finger nuclease.

## Competing interests

BLS is a founder and shareholder in a private biotechnology company (Pregenen Inc.) that creates and uses engineered homing endonucleases for genomic and cellular engineering applications.
